# Clinical analysis of molecular typing of 146 cases of endometrial carcinoma

**DOI:** 10.3389/fonc.2024.1482817

**Published:** 2025-01-20

**Authors:** Bo Zhang, Dan Zhou, Shuo Zhang, Jinbowen Yan, Qingwei Meng, Qiubo Lv

**Affiliations:** ^1^ Department of Obstetrics and Gynecology, Beijing Hospital, National Center of Gerontology, Institute of Geriatric Medicine, Chinese Academy of Medical Sciences, Dongdan, Beijing, China; ^2^ Chinese Academy of Medical Sciences and Peking Union Medical College, Beijing Hospital, Beijing, China

**Keywords:** endometrial cancer, molecular typing, clinical analysis, the prognosis, individual treatment

## Abstract

**Objective:**

To investigate the application of TCGA molecular typing in endometrial carcinoma, compare the relationship between molecular typing and clinicopathologic features, and provide a new idea for individual treatment of patients.

**Methods:**

A total of 146 EC patients who underwent surgical treatment and TCGA molecular typing in Beijing Hospital from December 2019 to March 2023 were collected. The clinicopathologic features, immunohistochemistry, and prognosis of the four TCGA molecular types were analyzed retrospectively.

**Result:**

Among the 146 patients with endometrial cancer (EC), 8 patients (5.5%) exhibited the POLE hypermutant type, 29 patients (19.9%) displayed the MSI-H type, 94 patients (64.4%) presented the low copy-number type, and 15 patients (10.3%) manifested the high copy-number type. A comparative analysis of the four TCGA types and age yielded statistically significant results (p = 0.012). Notably, significant associations were observed between menopausal status, the expression of ER, PR, and the four TCGA types. However, no significant difference was observed in CA125 levels before surgery among the four TCGA types (p = 0.587). There were significant differences observed among the four TCGA types and pathological types, pathological grades, FIGO stage, lymph node metastasis, and LVSI. The progression-free survival (PFS) rates of patients with POLE hypermutation, MSI-H type, CNL type, and CNH type were 100%, 100%, 93.62%, and 73.3%, respectively. There was a statistically significant difference between the four groups(p=0.006). POLE mutant and MSI-H type patients have higher PFS, while high copy type patients have the lowest.

**Conclusions:**

TCGA molecular typing has feasibility and application value in the clinical application of endometrial cancer, and has a certain predictive effect on the prognosis of EC patients. It has a certain guiding significance for the individual treatment of patients with endometrial cancer.

## Introduction

Endometrial cancer (EC) is the prevailing neoplasm affecting the female reproductive system (1), with an estimated annual incidence of approximately 382,000 new cases and a global mortality rate of 90,000 deaths (2).

EC is a class of heterogeneous malignant tumors, most patients have a better prognosis, but a small number of patients even if early detection also poor prognosis (1). Therefore, how accurately identifying the molecular biology characteristics of EC and targeted treatment has important clinical significance. Non-endometrioid ECs make up the majority of Type II ECs. They are more common in elderly women and are usually hormone-independent. This is a less common group (10–20%) but is associated with a higher risk of disease recurrence and a poor prognosis (25–60% 5 years OS rate) (1).

In 2013, The Cancer Genome Atlas (TCGA)transcriptomics and proteomics to 373 EC samples (including 307 ECCs, 53 SEC cases, and 13 hybrids). According to the molecular characteristics of EC, four molecular subtypes of POLE hypermutant type (7%), MSI-H (28%), CNL type (39%), and CN-H type (26% slurry) were proposed (3).

The molecular classification scheme provided prognostic insights, with the POLE hypermutant group exhibiting the most favorable progression-free survival (4), the CNH group displaying the poorest prognosis, while the MSI-H and CNL groups demonstrated intermediate progression-free survival rates (5).

This study constitutes a real-world investigation wherein we gathered clinical cases of endometrial cancer from Beijing Hospital to conduct a retrospective analysis. Our primary objective was to examine the epidemiological and clinical pathological characteristics associated with various molecular subtypes and assess the concordance between molecular and pathological classifications and their respective prognostic implications.

## Materials and methods

A total of 146 endometrial cancer (EC) patients who underwent surgery and TCGA molecular subtyping at Beijing Hospital between December 2019 and March 2023 were included in this study. Clinicopathological data were obtained from the medical record management system, encompassing patient age, menopausal status, pathological type and grade, surgical stage, lymphovascular space invasion (LVSI), preoperative serum CA125 levels, lymph node metastasis, and immunohistochemical markers, including estrogen receptor (ER) and progesterone receptor (PR) status.

The inclusion criteria were defined as follows: (1) a pathologically confirmed diagnosis of primary EC (endometrioid, clear cell, serous, or mixed; any grade, any FIGO stage); (2) performed TCGA molecular typing; (3) the availability of clinical information (baseline information, clinicopathologic features, and at least 1 month of follow-up).The exclusion criteria were as follows: (1) uterine sarcomas, (2) conservative surgery (any treatment option not including total hysterectomy and bilateral salpingo-oophorectomy for fertility purposes), and (3) any other malignancy present in the previous 5 years or synchronously.

Patients were followed up every 3 months for the first 2 years after completing treatment and every 6 months for the subsequent 3 years. Follow-up assessments primarily included imaging studies, such as pelvic and abdominal ultrasound or computed tomography (CT), as well as serum tumor marker evaluations. The follow-up period extended until June 31, 2023, with a median follow-up duration of 21 months (range: 2–59 months). The follow-up rate was 100%.

**Figure 1:**
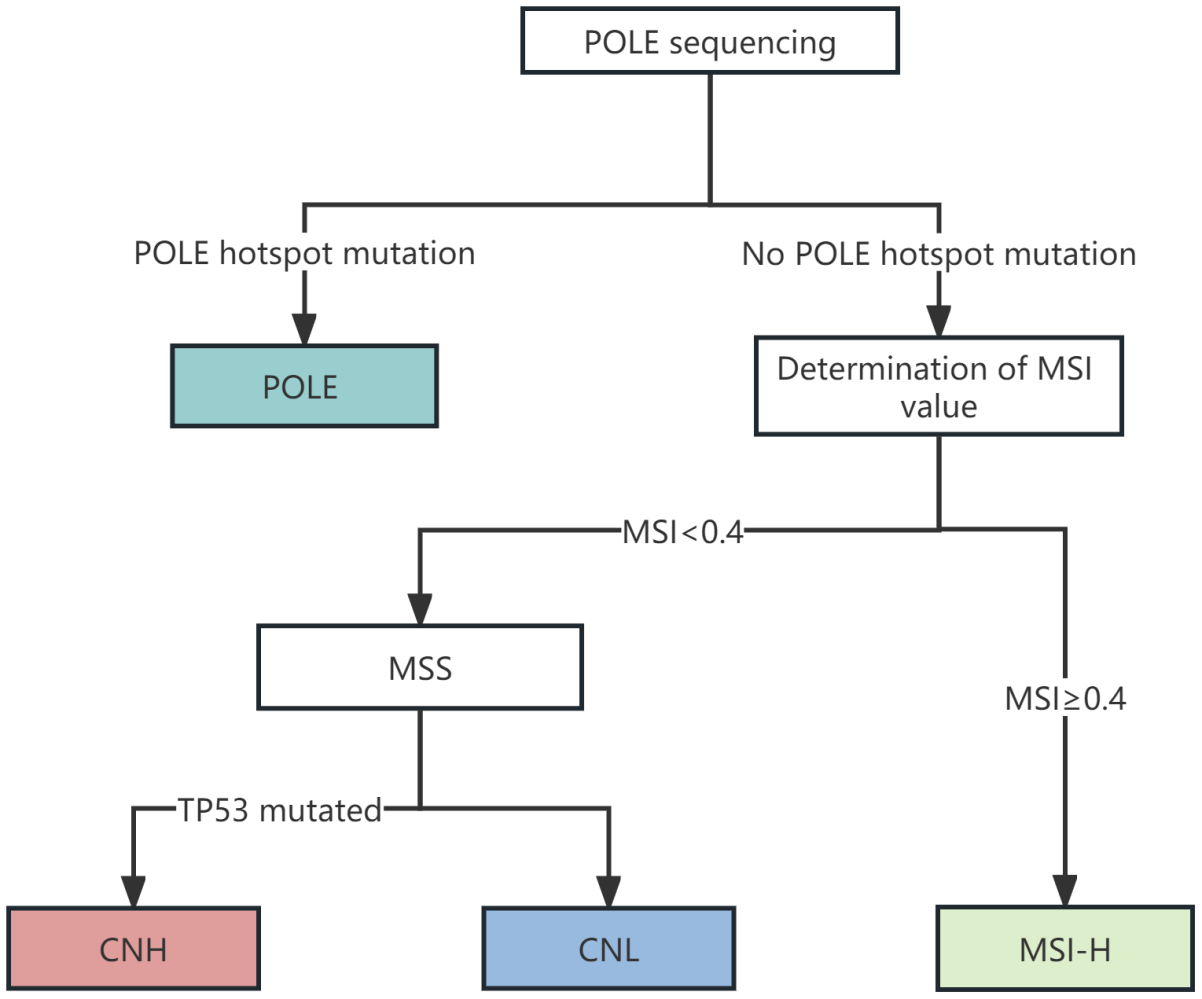
Flow chart of molecular typing of EC Patient Cancer Genome Atlas (TCGA).

High-throughput sequencing technology was used for gene detection. According to the 2013 TCGA molecular typing method, EC patients were divided into 4 types: The mutation status of the POLE gene is detected. If the POLE gene is mutated, it is determined to be POLE hypermutant; In POLE gene wild-type samples, microsatellite instability (MSI) value ≥0.4 was determined to be MSI-H type; In patients with microsatellite stability, TP53 gene mutation detection method was used instead of copy number or immunohistochemical detection method. If a TP53 gene mutation occurred, it was judged as a high copy type, and if no mutation occurred, it was judged as a low copy type (**Figure 1**).

SPSS 24.0 software was utilized for statistical analysis. The clinical characteristics, pathological features, and prognoses of cases across different molecular subtypes were analyzed. Survival curves were generated using the Kaplan-Meier method, and a P value of <0.05 was considered statistically significant.

## Results

A total of 146 patients with endometrial cancer (EC) were included in this study. The median age of the patients was 59 years (range: 32–88 years), with 46 patients (31.5%) aged over 65 years and 100 patients (68.5%) aged 65 years or younger. Among the 146 patients, 108 were postmenopausal. All patients underwent surgical treatment, with 98 undergoing open surgery and 48 undergoing laparoscopic surgery.

Among the 146 EC patients, 8 patients had POLE hypermutant type, 29 patients had MSI-H type, 94 patients had low copy type, and 15 patients had high copy type.

**Figure 2:**
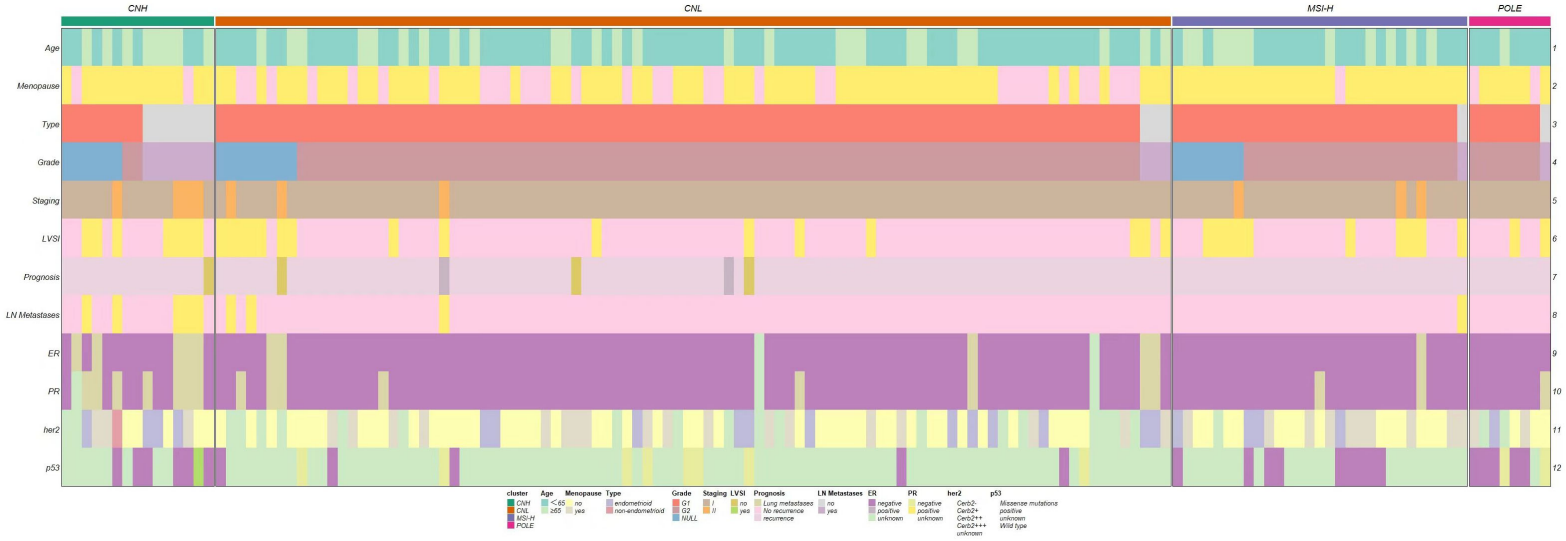
Comparison of different clinicopathological features in EC by 4 TCGA molecules.

No statistically significant difference was observed between the four TCGA types and CA125 levels before surgery (p=0.587). However, comparative analysis across the four TCGA types and age yielded statistically significant results (p = 0.012). Additionally, significant associations were found between menopausal status (p=0.002), expression of ER (p=0.046), PR (p=0.001), and the four TCGA types. Furthermore, significant differences were observed among the four TCGA types and pathological types (p<0.001), pathological grades (p<0.001), FIGO stage (p=0.013), lymph node metastasis (p=0.005), and LVSI (p=0.03) (**Table 1**, **Figure 2**).

**Table 1:** Comparison of different clinicopathological features in EC by 4 TCGA molecules (p<0.05 was considered statistically significant).

Category	Total Number	Pole Mutant	MSI-H Type	Low CN Type	High CN Type	χ^2^	p
Age	146					10.985	0.012
≥ 65	46	1	11	26	8		
< 65	100	7	18	68	7		
Menopausal status	146					14.310	0.002
+	108	6	28	61	13		
–	38	2	1	33	2		
Pathological types	146					21.184	<0.001
Endometrioid carcinoma	134	7	28	91	8		
Non Endometrioid carcinoma	12	1	1	3	7		
Pathological grades	134					21.493	<0.001
G1-G2	112	7	20	83	2		
G3	22	0	8	8	6		
FIGO Stage	146					9.404	0.013
I-II	136	8	26	91	11		
III-IV	10	0	3	3	4		
CA125	146					1.930	0.587
≥ 35	32	1	8	18	5		
< 35	114	7	21	76	10		
ER expression	146					9.444	0.014
+	134	8	28	88	10		
–	12	0	1	6	5		
PR expression	146					14.134	0.001
+	127	7	27	86	7		
–	19	1	2	8	8		
LVSI	146					8.413	0.030
+	35	2	10	16	7		
–	111	6	19	78	8		
lymph node metastasis	146					11.251	0.005
+	10	0	1	4	5		
–	136	8	28	90	10		

Statistical analysis revealed discrepancies between the MSI-H group classified by high-throughput sequencing and the immunohistochemical results. Among the 29 patients in the MSI-H group, immunohistochemistry indicated positive expression of MLH1, MSH2, MSH6, and PMS2 in 7 patients. Notably, negative expression of the PMS2 protein was the most frequently observed abnormality (**Table 2**).

**Table 2:** Expression of MLH1, MSH2, MSH6 and PMS2 in EC with four TCGA molecular types.

Category	Total Number	Pole Mutant N (N%)	MSI-H Type N (N%)	Low CN Type N (N%)	High CN Type N (N%)
MLH1 Expression	146				
+	126	8 (6.4)	11 (8.7)	92 (73.0)	15 (11.9)
–	20	0 (0)	18 (90.0)	2 (10.0)	0 (0)
PMS2 Expression	146				
+	124	7 (5.6)	10 (8.1)	92 (74.2)	15 (12.1)
–	22	1 (4.5)	19 (86.4)	2 (9.1)	0 (0)
MSH2 Expression	146				
+	143	8 (5.6)	26 (18.2)	94 (65.7)	15 (10.5)
–	3	0 (0)	3 (100)	0 (0)	0 (0)
MSH6 Expression	146				
+	144	8 (5.6)	27 (18.7)	94 (65.3)	15 (10.4)
–	2	0 (0)	2 (100)	0 (0)	0 (0)

Patients were followed up every 3 months for 2 years after completing treatment and every 6 months for 3 years after that. The follow-up period was up to May 31, 2023. The median follow-up time of 146 patients was 21 months (range: 2-59 months). The follow-up rate was 100%.

**Figure 3:**
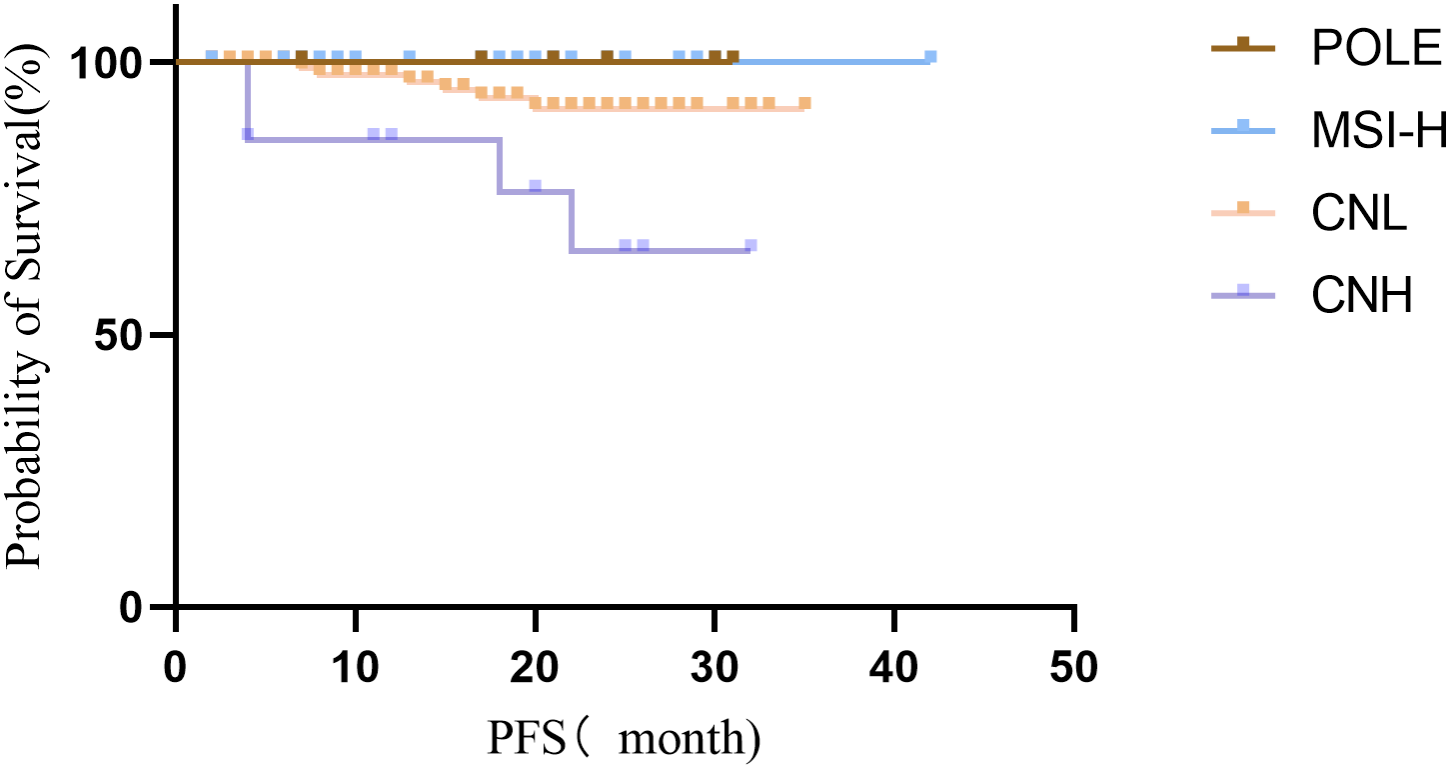
Survival curves of patients with endometrial cancer classified by the Four TCGA.

None of the 146 EC patients died during the follow-up period. A total of 136 patients (93.15%) remained progression-free, while 10 patients (6.85%) experienced recurrence, including 4 cases in the CNH group and 6 cases in the CNL group. The progression-free survival (PFS) rates for patients with POLE hypermutation, MSI-H type, low copy number type, and high copy number type were 100%, 100%, 93.62%, and 73.3%, respectively. The differences in PFS among the four groups were statistically significant (P = 0.006). Patients with POLE hypermutation and MSI-H subtypes demonstrated the highest PFS rates, whereas those with the high copy number subtype exhibited the lowest (**Figure 3**).

## Discussion

The traditional binary classification method has been widely used in clinical practice for high-risk endometrial cancer prediction and decision-making. However, its predictive ability is limited in clinical applications. In 2013, the Cancer Genome Atlas (TCGA) Research Network of the United States introduced a more advanced classification system for endometrial cancer (EC), dividing it into four categories based on gene proteomics. TCGA molecular typing has significantly enhanced the accuracy and reproducibility of EC diagnosis and provides valuable insights for assessing prognosis and guiding treatment decisions. This study aimed to preliminarily explore the clinical application value of TCGA molecular typing through a retrospective analysis of the relationship between clinicopathological features, immune-related molecular characteristics, and TCGA molecular subtypes in EC patients.

POLE is the catalytic subunit of DNA polymerase in the process of DNA replication and repair, and the mutation of POLE exonuclease will lead to this type. The mutation hot spots were P286R and V411L (6). Accounts for 5% to 10% of all endometrial cancers (7). In this study, there were only 8 cases in the POLE group, all of which were stage I endometrioid carcinoma, and no recurrence was observed during the follow-up period, which was consistent with literature reports as a whole (5). The prognosis of the POLE group was better, which might be related to a large number of tumor mutant genes, the increased number of tumor-expressed proteins (neoantigens), and the increased immune response. In addition, other gene mutations were often associated with the POLE group, such as PTEN, PIK3RI, PIK3CA, KRAS, etc (6).

“Microsatellite sequence” refers to short tandem repeats, which can exist widely in coding and non-coding regions (8). When mismatch repair genes are defective, MSI-H can appear. Compared with the microsatellite stable type, this type has a higher mutation load, including mutations of PTEN, KRAS, and RID 1A (6). It accounts for about 30% of EC, most of which are endometrioid carcinoma, and are of high grade. In this study, a total of 29 patients with MSI-H were included, including 1 patient with mixed-type cancer, 8 patients with high-grade, 15 patients with medium-grade, and 5 patients with low-grade endometrial carcinoma among the 29 cases. During the follow-up period, there was no recurrence, and the prognosis was relatively good, which was between the POLE hypermutant type and low copy type. deficient MSI-H (dMMR) EC has been added to the NCCN guidelines as a treatment option for MSI-H and deficient MMR (DMMR) EC, which has been found to have a high TMB based on data from the TCGA project and existing studies (9). This suggests that patients with MSI-H may be more likely to benefit from immunotherapy by using TMB to identify the tumor load. In addition, we statistically found that the MSI-H group classified by high-throughput sequencing did not fully match the immunohistochemical results. Immunohistochemical results of 7 of the 29 patients showed positive MLH1, MSH2, MSH6, and PMS2 expression. Among the four genes, the negative rate of PMS2 was the highest. This may be related to the false negative expression of PMS2.

CHL is characterized by low-frequency mutations and mainly consists of microsatellite-stabilized ECs. TCGA data show that low copy type accounts for about 39% of the total (10). 60% of low-grade and 8.7% of high-grade endometrioid cancers were of this type, as were 25% of mixed and 2.3% of serous cancers (11). In this study, 94 cases of low copy type were classified as stage I-II, with mainly low and medium grade and only 5 cases of high grade. In this study, the lymph node metastasis rate, LVSI positive rate, and prognosis of the CNL group were all between the CNH group and POLE group. Except for POLE mutations, MSI-H, and p53 mutations, all patients with endometrial cancer were classified as CNL. Therefore, the guiding significance of the CNL group for treatment is not clear. In this study, one of the recurrent patients in CNL had a mutation in the CTNNB1 gene (12). This suggests that combined with immunohistochemistry and genetic testing, more guiding molecular markers can be found to provide more effective help to patients (13).

CNH is characterized by a low mutation rate and high-frequency copy number changes, accounting for about 26% of the total EC (14). Histologically, this type is mainly composed of serous EC, and a quarter of high-grade endometrioid carcinomas belong to this type. TP53 mutation was the most common type of EC (mutation rate was 91.7%). In this study, there were 15 cases in the CNH group, In addition, among the 4 groups, this type of patient had the latest surgical pathological stage, the highest pathological grade, and the worst prognosis. However, it is gratifying that 25% of EC specimens of this type were also found to have the amplification of the ERBB2 gene, which can also guide clinical detection of HER2 molecular in these patients for targeted therapy (4).

The TCGA molecular typing sequencing technology offers enhanced accuracy and provides several benefits, including the evaluation of prognosis and guidance for individualized therapy. Moreover, it largely mitigates the challenges associated with the inconsistent diagnosis of histological types (15). However, sequencing technology is hindered by its high cost, complexity, and limited clinical applicability. Additionally, its use is restricted to fresh tissue samples, excluding biopsy, curettage, and other specimens. Consequently, its application is constrained, and it cannot effectively serve as a reference for preoperative patient management. Therefore, in recent years, researchers are committed to exploring molecular typing methods that are simple, clinically practical, and can be routinely carried out in pathology departments. Talhouk et al. invented ProMisE (Proactive Molecular Risk Classifier for Endometrial Cancer) model in 2015. In this improved method, the dMMR group was determined by immunohistochemical detection of MMR proteins (MLH1, MSH2, MSH6, PMS2), and the POLE EDM mutation group was determined by sequencing. The third step was to use the p53 immunohistochemical method instead of copy number status detection to determine the p53 mutation group and the p53 wild group to reproduce the TCGA gene grouping (16). This method has lower cost and higher efficiency and is suitable for biopsy and paraffin-embedded specimens. Moreover, the diagnostic specimens of this classification method have high consistency with the pathological diagnosis of final hysterectomy specimens. However, at present, ProMisE molecular classification only replicates the prognostic survival curve of TCGA molecular typing. In the future, various clinicopathological indicators, histological morphology, and other molecular parameters of patients should be selectively added to enhance the accuracy of grouping and expand its application range.

The purpose of TCGA molecular typing is to refine the risk stratification of endometrial cancer, thereby improving treatment strategies and reducing unnecessary postoperative adjuvant therapies and medical expenses, without compromising treatment outcomes or patient prognosis. While the current molecular typing method offers more accurate prognostic predictions than traditional pathological typing, its benefits are limited to specific patient populations. The study by Carlo Ronsini et al. introduces a novel biomarker for identifying endometrial cancer risk in patients with abnormal uterine bleeding during menopause. By combining systemic inflammatory indices with endometrial thickness, the SIR-En index effectively differentiates between endometrial hyperplasia and carcinoma in menopausal women with abnormal uterine bleeding. (17). The study by Irene Iavarone et al. investigated the distribution and regulation of differentially expressed miRNAs (DEMs) and extracellular vesicle-derived substances in women with endometrial cancer. The upregulated molecules derived from extracellular vesicles (EVs) present potential as biomarkers for the early detection of endometrial cancer (18). The study by Camilla Nero et al. confirms that the molecular features included in the risk assessment system represent a paradigm shift in the prognostic classification of patients with endometrial cancer (EC). Specifically, patients with the NSMP (non-specific molecular profile) subtype can be more effectively stratified based on factors such as lymphovascular space invasion (LVSI) status, L1CAM expression, and CTNNB1 mutations (18). Further research is needed to identify new molecular markers or combine different molecular predictors in order to develop more accurate molecular models that can better guide clinical practice.

In summary, a total of 146 EC cases were gathered and assessed utilizing the TCGA molecular typing theory. The clinicopathological characteristics and prognostic relationship of each subtype were summarized and combined with literature analysis. Molecular typing of EC provides more objective and accurate prognostic information for the clinic and guides the clinical diagnosis and treatment of patients to a certain extent. Nevertheless, given the constraints of conventional TCGA molecular typing, it remains imperative to investigate alternative methods that are both cost-effective and suitable for routine clinical application.

This study has several limitations that must be acknowledged. First, as a retrospective analysis relying on existing clinical data, it is subject to potential bias and limited control over confounding factors, which may affect the reliability of the conclusions. Second, the study primarily focuses on progression-free survival (PFS).Due to the limitation of follow-up duration, long-term overall survival (OS) data has not yet been obtained. We plan to further improve the OS dataset in future studies. Third, while the TCGA subtypes provide a broad classification framework, the genetic heterogeneity within each subtype was not explored. Our subsequent research is currently focused on conducting further subgroup analyses of gene mutation subtypes. Additionally, the absence of an external validation cohort restricts the generalizability of our findings across diverse populations. We are actively seeking collaborations with other institutions and aim to incorporate multi-population datasets to ensure the generalizability of our findings across diverse settings and patient cohorts. Lastly, the study did not integrate other genomic features (e.g., mutations, copy-number variations) or assess the impact of molecular subtyping on personalized treatment strategies. Future studies should address these gaps to enhance clinical applicability.

## Data Availability

The original contributions presented in the study are included in the article/supplementary material. Further inquiries can be directed to the corresponding author.
